# Antibody responses after sequential vaccination with PCV13 and PPSV23 in patients with moderate to severe plaque psoriasis under immunosuppressive therapy

**DOI:** 10.1128/mbio.00482-24

**Published:** 2024-06-04

**Authors:** Lorena Helmer, Lukas van de Sand, Thea Wojtakowski, Mona Otte, Oliver Witzke, Wiebke Sondermann, Adalbert Krawczyk, Monika Lindemann

**Affiliations:** 1 Department of Infectious Diseases, University Hospital Essen, University Duisburg-Essen, Essen, Germany; 2 Institute for Transfusion Medicine, University Hospital Essen, University Duisburg-Essen, Essen, Germany; 3 Department of Dermatology, Venereology and Allergology, University Hospital Essen, University Duisburg-Essen, Essen, Germany; 4 Institute for Virology, University Hospital Essen, University Duisburg-Essen, Essen, Germany; Fondazione Biotecnopolo di Siena, Siena, Italy

**Keywords:** *Streptococcus pneumoniae*, vaccination, psoriasis, sequential vaccination, serotype-specific immunity, pneumococcal antigens

## Abstract

A crucial step in lowering the risk of invasive pneumococcal illness in high-risk populations, such as individuals with plaque psoriasis, is pneumococcal vaccination. The serologic response to the sequential vaccination with Prevenar 13 (PCV13) and Pneumovax 23 (PPSV23) in psoriasis patients under immunosuppressive therapy is still poorly characterized despite national recommendations suggesting vaccination for immunocompromised patients. In this prospective study, we investigated the serological response in 57 patients under active systemic treatment for moderate to severe plaque psoriasis who underwent sequential vaccination with PCV13 followed by PPSV23. Our analysis focused on global and serotype-specific anti-pneumococcal antibody responses over a 7-month period post-vaccination. Our findings reveal a robust serological response in patients with plaque psoriasis under systemic therapy. When comparing our results with a cohort of kidney transplant recipients who completed a similar sequential vaccination protocol, psoriasis patients showed higher antibody concentrations. In psoriasis patients, the mean levels of all global antibody classes tested (IgG, IgG2, IgA, IgM) increased more than 4-fold (*P* < 0.0001) and serotype-specific antibodies more than 1.9-fold (*P* < 0.01). In addition to providing strong evidence of the safety and effectiveness of sequential pneumococcal vaccination in individuals with plaque psoriasis, our work sheds light on the complex interactions that exist between immunosuppressive treatment, vaccination schedule, and antibody responses in various risk groups.

**IMPORTANCE:**

To protect against severe courses of infection with *Streptococcus pneumoniae*, the national guidelines recommend sequential vaccination for these patients. However, there are only studies on the efficacy of a single administration of these vaccines in this particular risk group. The immunological responses to the vaccine were correlated with clinical patient data. In summary, our study shows for the first time that sequential vaccination is immunogenic in patients with moderate to severe plaque psoriasis.

## INTRODUCTION

Psoriasis is a chronic, immune-mediated inflammatory disease that leads to typical erythematous, scaly skin plaques. It is one of the most common inflammatory dermatoses, affecting approximately 2% of the German population (1). Based on current scientific knowledge, the pathophysiology of psoriasis involves an excessive release of pro-inflammatory molecules from the acquired immune system such as tumor necrosis factor alpha (TNF-α) and interleukins (IL), especially IL-17 and IL-23 (2).

Mild psoriasis is usually defined as psoriasis area and severity index (PASI) of ≤10 and/or body surface area (BSA) of ≤10% and dermatology life quality index (DLQI)of ≤10, and moderate to severe psoriasis is usually defined as PASI or BSA of >10 and DLQI of >10. Special clinical situations can change mild psoriasis to moderate to severe, including involvement of visible areas or severe nail involvement (3).

Systemic therapy options for patients with moderate to severe plaque psoriasis include immunosuppressive and immunomodulatory therapies such as fumaric acid esters, methotrexate (MTX), the small molecule apremilast (phosphodiesterase-4 inhibitor), and various biologicals. The first group of biologics available for the treatment of psoriasis was TNF-α inhibitors such as adalimumab, etanercept, infliximab, and certolizumab. The newer biologics target specifically interleukin IL-12/23 (ustekinumab), IL-23 (guselkumab, tildrakizumab, and risankizumab), or IL-17 (secukinumab, ixekizumab, bimekizumab, and brodalumab) (4, 5).

Patients with psoriasis under immunosuppressive therapy are at an increased risk of infectious complications, including lower respiratory tract infections or pneumonia due to the impairment of the immune system caused by the therapy. This applies in particular to treatment regimens with MTX and TNF-α blockers (6). Pneumonia is the most common fatal infectious disease in industrialized countries. Pneumonia caused by *Streptococcus pneumoniae* (*S. pneumoniae*) can be particularly severe. *S. pneumoniae* is a gram-positive bacterium that colonizes the upper airway of humans and can cause local and systemic diseases. It remains the leading cause of community-acquired pneumonia worldwide (7). Patients with psoriasis who receive immunosuppressive therapy are at a higher risk for invasive pneumococcal disease, which can be prevented through vaccination.

The main virulence factor of *S. pneumoniae* is its capsule, which is composed of various polysaccharides. Currently, there are over 90 serotypes of pneumococci, but the majority of infections are caused by 23 of these serotypes (8). In clinical practice, two types of pneumococcal vaccines are used, such as the pneumococcal polysaccharide vaccine (PPSV) and the pneumococcal conjugate vaccine (PCV).

PPSV is a T-cell-independent type 2 antigen that induces IgG responses but does not generate memory B cells as well. In contrast, PCV is covalently conjugated to carrier proteins to enhance immunogenicity. The protein in PCV induces a T helper cell response, which can promote B-cell differentiation into antibody-producing plasma cells or memory B cells.

In Germany, high-risk groups, including psoriasis patients receiving immunosuppressive therapy, are recommended to receive a sequential administration of PCV13 followed by PPSV23 after 6–12 months. However, it is still unclear to what extent serological titers reflect protection against *S. pneumoniae* infection. To date, there has been limited research on the effectiveness of pneumococcal vaccination or sequential vaccination in patients with immunosuppression and immunosuppressive therapy, respectively, due to a chronic inflammatory disease such as psoriasis. Rákócz and Szekanecz analyzed the influence of different immunosuppressive drugs on the antibody response after PPSV23 or PCV13 administration in patients with autoimmune rheumatic diseases. They highly recommend the administration of PCV13 followed by PPSV23 (9). Additionally, clinical studies demonstrate the beneficial effects of sequential pneumococcal vaccination in solid-organ recipients (10, 11).

However, there are currently no data on the serological response in psoriasis patients who received a sequential vaccination with PCV13 and PPSV23. This study aims to investigate the serological immunogenicity and safety of sequential PCV13 and PPSV23 vaccination in patients with moderate to severe plaque psoriasis receiving immunosuppressive and immunomodulatory therapy, respectively. In addition to measuring a global pneumococcal antibody response, we also determined specific immune responses to six pneumococcal serotypes.

## MATERIALS AND METHODS

### Study population

This single-center study enrolled 57 individuals diagnosed with moderate to severe psoriasis between March 2020 and December 2020. Table 1 provides demographic details. The cohort consisted of 32 males and 25 females with a median age of 48 years (range: 18–79 years). The median time between initial diagnosis and study inclusion was 25 years.

**TABLE 1:** Basic patient characteristics

Parameter	Result
Median age (range), years^*a*^	48 (18–79)
Patient sex (male/female)	32/25
Median interval since first diagnosis (range), years	25 (1-50)
Immunosuppression, no.^*a*^	
MTX monotherapy ≥10 mg/week	7
TNF-α inhibitor (adalimumab/infliximab/etanercept/certolizumab), plus MTX co-medication max. 10 mg/week	18
IL-17 inhibitor (secukinumab/ixekizumab)	11
IL-12/23 inhibitor (ustekinumab)	8
IL-23 inhibitor (guselkumab/tildrakizumab)	13

^*a*^ At the time of first blood sampling.

All participants had reached legal age, provided informed consent, and underwent treatment according to one of the regimens specified in Table 1 due to moderate to severe forms of psoriasis as defined above. The systemic therapy had been administered for at least 1 month at the time of inclusion. Exclusion criteria included pregnancy, changes in therapy group during the study, and prior vaccination against *S. pneumoniae* within the previous 6 years.

**Fig 1:**
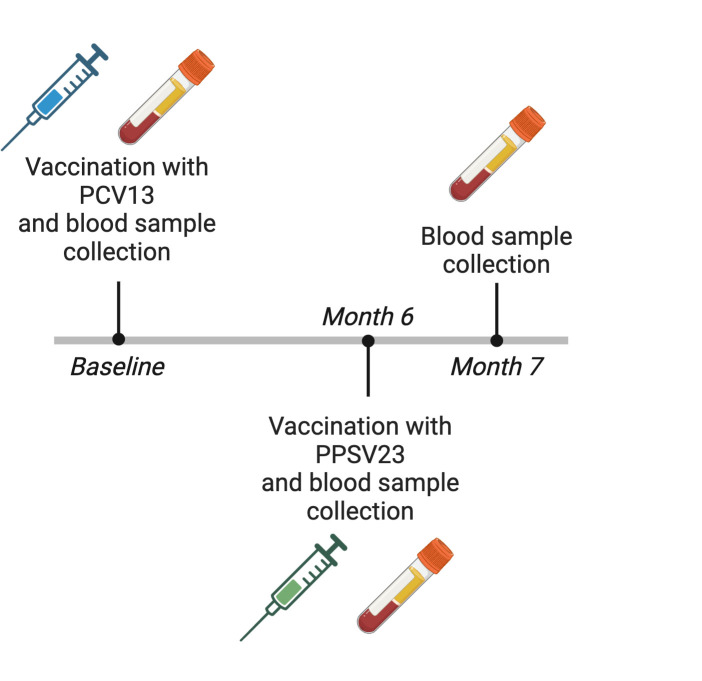
Study design. All patients with plaque psoriasis sequentially received a single dose of PCV13 and PPSV23. Blood samples were drawn at the indicated time points.

Figure 1 illustrates the successive inoculations of PCV13 and PPSV23 received by the subjects. Blood specimens were collected before each vaccination and at the conclusion of month 7 after the initial vaccination, corresponding to month 1 after the second vaccination. Clinical endpoints such as incidents of pneumonia were monitored until month 26 following the primary vaccination.

Potassium EDTA for the determination of leukocytes by routine laboratory measurements of patients was performed on the same day according to local standards using XN-1000 Pure (Sysmex, Norderstedt, Germany).

Ethical endorsement for the study was received from the institutional review board of the University Hospital Essen (19-8679-BO), and written consent was obtained from all participants. The study adhered strictly to the principles outlined in the Declarations of Helsinki and Istanbul, as well as their subsequent amendments.

### Vaccines

The Prevenar 13 vaccine contains polysaccharides corresponding to 13 serotypes, including serotypes 1, 3, 4, 5, 6A, 6B, 7F, 9V, 14, 18C, 19F, 19A, and 23F, as well as the cross-reactive component 197 derived from diphtheria toxin. Each dose contains 2.2 µg of each serotype, except for serotype 6B, which is administered at 4.4 µg per dose (0.5 mL).

The Pneumovax 23 vaccine is an unconjugated formulation that contains 25 µg of each of the 23 pneumococcal serotypes, including serotypes 1, 2, 3, 4, 5, 6B, 7F, 8, 9N, 9V, 10A, 11A, 12F, 14, 15B, 17F, 18C, 19F, 19A, 20, 22F, 23F, and 33F. Comprehensive vaccine details have been previously provided. Both vaccines were injected intramuscularly into the deltoid muscle (12).

### Control group

A cohort of vaccine-naïve healthy blood donors delineated in two antecedent studies that established reference values for IgG and IgG2 (13) and for IgA and IgM (14), respectively, served as a control group. The identical commercial enzyme-linked immunosorbent assay (ELISA) used to assess the current research population’s pneumococcal antibody levels was also applied to evaluate the historical control group (VaccZyme, The Binding Site, Schwetzingen, Germany).

The vaccination responses in psoriasis patients were also compared with data from a similar study in kidney transplant patients (KTP) carried out at our center (11). This study included 46 kidney transplant patients between November 2018 and October 2019. The age range was 22–76 years, with a median of 57 years. Thirteen patients were female, and 31 were male. The median interval between the last kidney transplantation and study inclusion was 38 months (range 3–338 months).

### Measurement of serotype-specific antibody concentrations

The concentrations of serotype-specific IgG antibodies against six distinct pneumococcal serotypes (2, 3, 6A, 9N, 11A, 14) were quantified using the enzyme-linked immunosorbent assay. The isotype-specific reference ELISA was conducted following the protocol outlined by the World Health Organization (WHO) (https://www.vaccine.uab.edu/uploads/mdocs/ELISAProtocol(007sp).pdf).

Briefly, 96-well microtiter plates (Greiner Bio-One, Frickenhausen, Germany) were initially coated with serotype-specific pneumococcal polysaccharide (Pn PS) antigen (ATCC, Manassas, VA, USA). The plates were then incubated at 37°C for 5 hours in a humid chamber. Before use, the plates were blocked with DPBS plus 1% (wt/vol) dried non-fat milk powder for 1 hour at room temperature.

Furthermore, patient serum samples and a human anti-pneumococcal reference serum (007sp) were pre-incubated before analysis with an absorbent containing cell-wall polysaccharide and pneumococcal type 22F capsular polysaccharide. Quality control serum was sourced from the Pneumococcal Reference Laboratory in Birmingham, AL, USA, and kindly provided by Mustafa Akkoyunlu. Following pre-incubation, both control and test sera were added to the plates in duplicate wells and incubated at room temperature. An anti-human IgG (gamma-chain specific) F(ab')2 fragment peroxidase was used to detect the antibodies bound to the plates for all IgG subclasses (A2290; Sigma-Aldrich, St. Louis, MO, USA).

The reaction was stopped by adding a sulfonic acid stop solution to the TMB ELISA substrate (Thermo Fisher Scientific, Cleveland, OH, USA). Optical density of each well was measured at 405 and 690 nm (reference) using the Tristar 3 multimode ELISA plate reader (Berthold Technologies, Bad Wildbad, Germany). The serum antibody concentrations were calculated using log-linear regression analysis with GraphPad Prism 9 software (GraphPad Prism Software, San Diego, CA, USA). The detection limit of this assay was 0.01 mg/L.

### Determination of global antibody responses against pneumococci

Four different commercial ELISA formats (VaccZyme, The Binding Site Group Ltd., Birmingham, UK) were used to detect antibodies against *S. pneumoniae*. These formats could identify IgG, IgG2, IgA, and IgM antibodies against the same 23 pneumococcal serotypes included in the PPSV23 vaccine. In the following, the assay is called global pneumococcal ELISA. The assay was performed following the manufacturer’s instructions.

### Statistical analysis

The data were analyzed using either GraphPad Prism or IBM SPSS Statistics version 25 (Armonk, NY, USA). The normal distribution of the data set was assessed using the Shapiro-Wilk test. As non-normal distribution was observed in several variables, non-parametric tests were applied for subsequent data analysis. The Kruskal-Wallis test, followed by Dunn’s multiple comparisons test, was used to assess ELISA responses across various time points. The Mann-Whitney *U* test was used to compare continuous variables, and the unpaired Student’s *t* test was used to determine if the mean values differed between the current and previous study groups.

The correlation between numerical variables was determined using the two-tailed Spearman test. Multivariate analysis was conducted using logistic regression to investigate the impact of medication on pneumococcal antibodies. Results with a *P*-value less than 0.05 were considered statistically significant.

## RESULTS

### Clinical course of the study population and safety

During the study period, all patients were clinically examined until 12 months after receiving the first vaccination. There were no cases of hospitalized pneumonia caused by *S. pneumoniae*. However, one patient died after COVID-19 without the identification of bacterial superinfection or a specific bacterial pathogen. Twenty-one patients were diagnosed with psoriatic arthritis at the time of study inclusion. On average, clinical disease activity during the study period was rather low (mean PASI month 0: 2.25; mean PASI month 6: 2.03). Based on PASI and DLQI, as described in the introduction, no progress of psoriasis or related illnesses was observed in our cohort during the 26-month follow-up period after the initial vaccination with PCV13.

The subjects had been on their current systemic anti-psoriatic therapy for an average of 26 months. None of the patients received other immunosuppressive therapies for any indication other than psoriasis. Five subjects were diagnosed with lymphopenia during psoriasis therapy after taking fumaric acid esters. However, with a mean value of 1.23 /nL, the lymphocyte count in our subjects remained stable at the time of vaccination.

Reactions to vaccination, including erythema, swelling, injection site pain, and general symptoms such as subfebrile temperatures, headache, and local musculoskeletal pain, were classified as mild. In the entire study population, only those mild reactions to the vaccination were observed. None of the patients exhibited any serious complications or adverse effects attributable to the vaccination. There were no instances of safety-related withdrawals nor vaccination-associated deaths documented throughout the course of the study.

### Pneumococcal antibodies prior to and post vaccination

**Fig 2:**
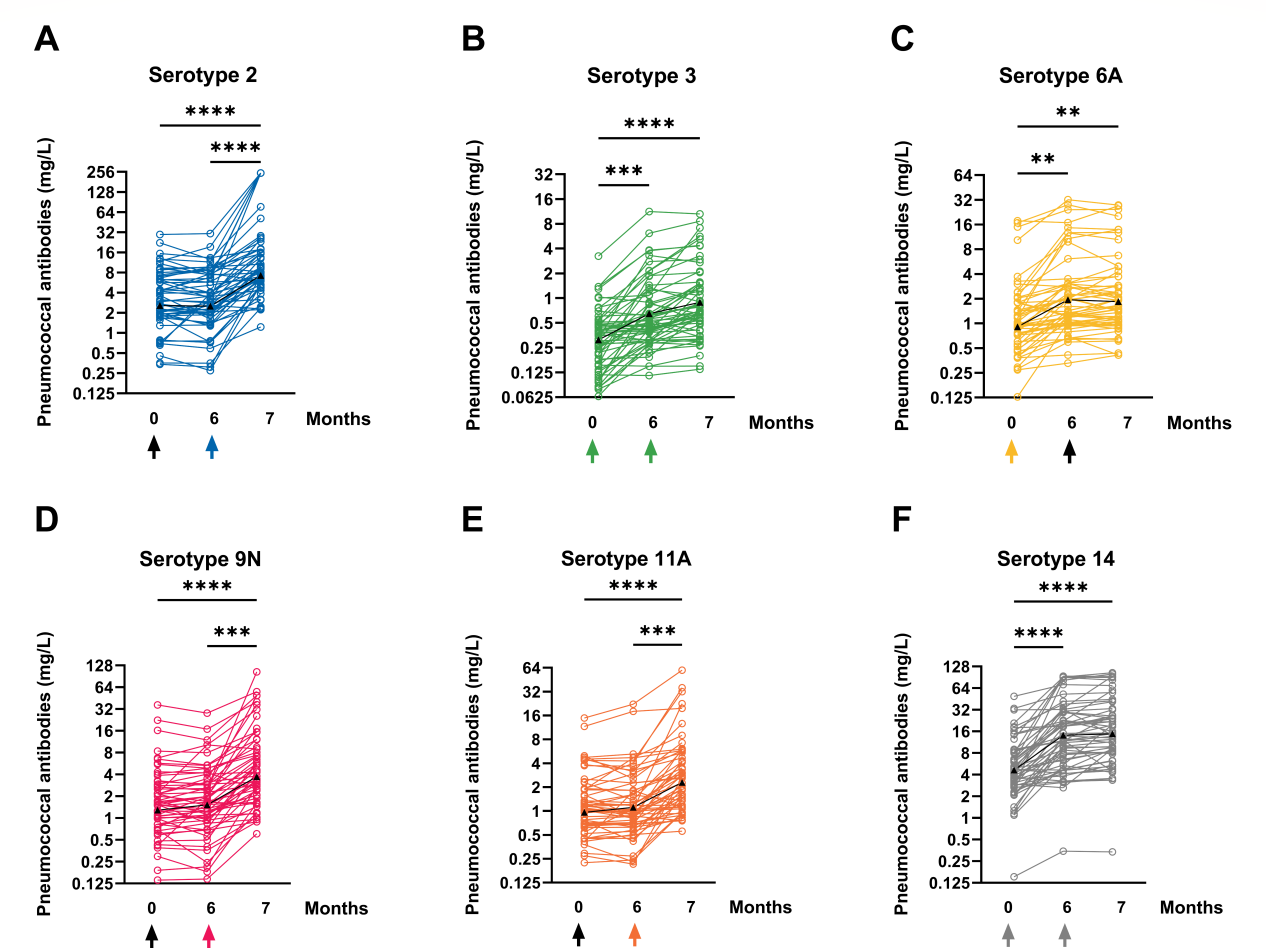
The graphs in panels A–F display the changes in serotype-specific pneumococcal antibodies for six different serotypes, including 2, 3, 6A, 9N, 11A, and 14, before and after vaccinations with PCV13 (at month 0) and PPSV23 (at month 6). The time of vaccination is indicated by an arrow, and the colored arrows indicate that the vaccine contains the corresponding serotype. The geometric mean values are represented by a black line with triangles, and the results are displayed on a logarithmic scale (log2). The data from 57 psoriasis patients were analyzed using the Kruskal-Wallis test, with Dunn’s multiple comparisons test. **P < 0.01, ***P < 0.0005, ****P < 0.0001.

We analyzed 171 sera from 57 patients at 3 measurement times using a standardized WHO pneumococcal ELISA. It was tested against the six serotypes 2, 3, 6A, 9N, 11A, and 14 (Fig. 2). Antibody concentrations were measured for all serotypes over the 7-month measurement period (from M0 to M7). Antibodies to all tested serotypes increased significantly at month 7 as compared to baseline (*P* < 0.0001). The highest antibody concentration within the three measurement times was always measured at M7. There was no significant decrease in antibody concentrations over the course of all measurements.

Between M0 and M6, there was no significant increase in antibody concentrations for serotypes 2, 9N, and 11A, which are exclusively contained in the second vaccine PPSV23. The effects of this vaccine can only be determined at M7.

**Fig 3:**
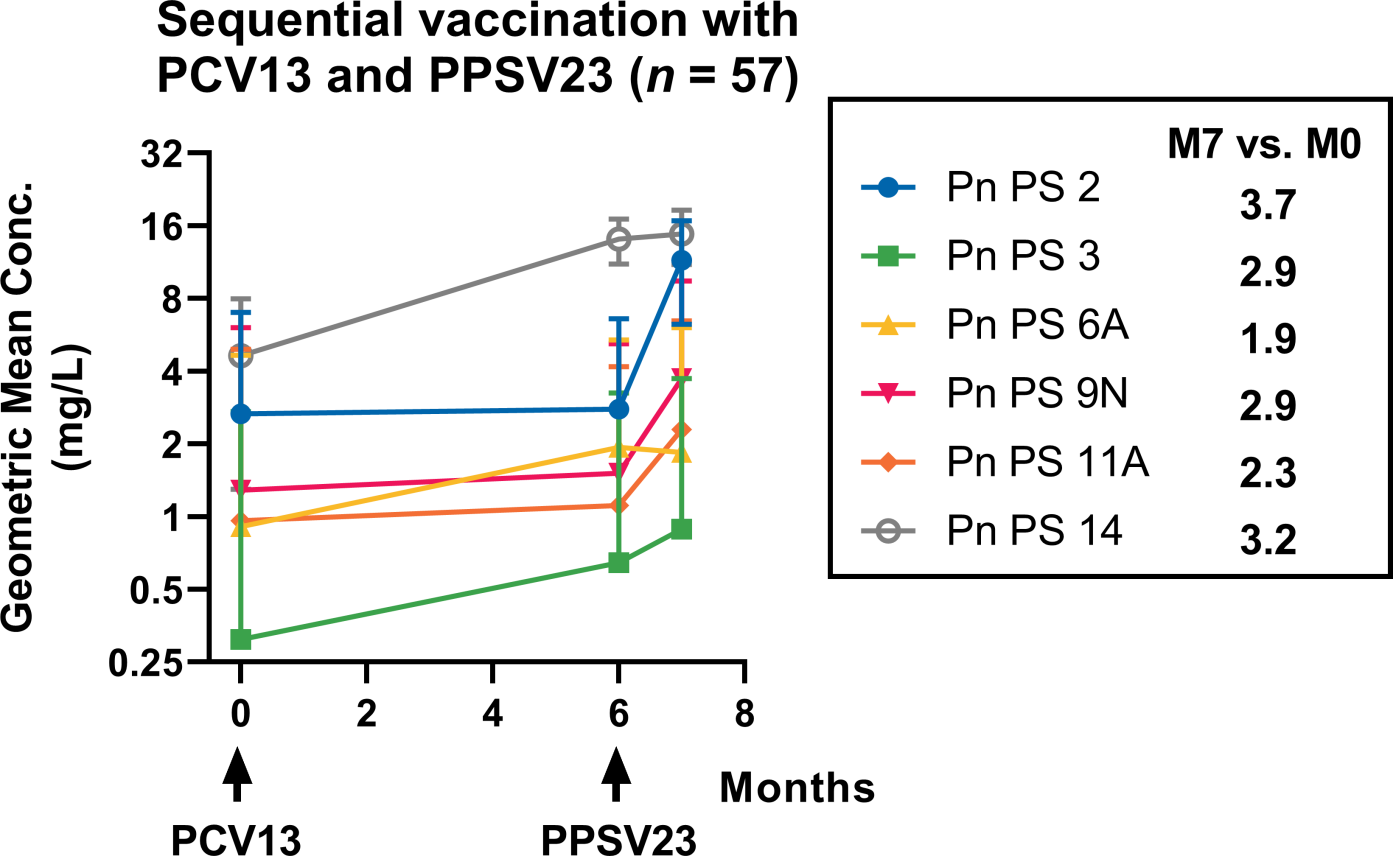
Concentrations of serotype-specific pneumococcal antibodies in 57 plaque psoriasis patients were tracked over the course of 7 months after their first vaccination. The patients’ antibody levels were measured before their first vaccination with PCV13 (M0), before their second vaccination with PPSV23 (M6), and at month 7 after the first vaccination, i.e., month 1 after the second vaccination (M7). The results are presented as the geometric mean concentration and geometric standard deviation factor, and the time of vaccination is marked with an arrow.

The lowest geometric mean concentration (GMC) at time point M7 after sequential vaccination with PCV13 and PPSV23 was measured for serotype 3. Despite being vaccinated against serotype 3 twice as it is contained in both vaccines PCV13 and PPSV23, mean antibody concentrations for this serotype always remained at low levels (M0: 0.3 mg/L, M6: 0.6 mg/L, M7: 0.9 mg/L) (Table 2). The low total levels notwithstanding, this serotype exhibited a significant mean increase from M0 to M6 (2.9-fold, *P* < 0.0005) (Fig. 3). The second vaccination with PPSV23 resulted in only minor effects on antibody levels after 7 months despite the inclusion of serotype 3.

**TABLE 2:** Serologic responses to sequential pneumococcal vaccination^*a*^

Time point	Serotype					
	2	3	6A	9N	11A	14
Month 0	2.8 (1.8–6.7)	0.3 (0.2–0.5)	1.0 (0.5–1.8)	1.5 (0.8–2.8)	1.1 (0.6–1.8)	4.8 (2.8–8.4)
Month 6	2.9 (1.9–7.5)	0.6 (0.3–1.1)	1.9 (1.0–3.0)	1,6 (0.9–3.2)	1.1 (0.7–1.9)	13.4 (6.1–29.3)
	*1.0* *(0.8–1.2)*	*2.7* *(1.1–3.4)*	*1.9* *(1.1–2.8)*	*1.0* *(0.9–1.3)*	*1.0* *(0.9–1.1)*	*2.8* *(1.5–4.8)*
Month 7	10.4 (5.3–17.8)	0.9 (0.5–1.5)	2.0 (1.0–3.1)	4.3 (1.8–9.1)	2.5 (1.1–4.9)	15.5 (8.3–28.1)
	*3.7* *(1.7–6.4)*	*2.9* *(1.6–5.1)*	*1.9* *(1.2–3.2)*	*2.9* *(1.5–4.0)*	*2.3* *(1.3–3.4)*	*3.2* *(1.7–4.6)*

^*a*^ Geometric mean concentrations (IQR) (mg/L) are in roman type; geometric mean fold increases (IQR) are in italics; *n* = 57.

The lowest total increase and second lowest total antibody concentration were analyzed for serotype 6A (1.9-fold, *P* < 0.01), which is only included in the PCV13 vaccine and is therefore only vaccinated at M0. This serotype showed a 1.9-fold increase from M0 to M6 (M0: 1.0 mg/L, M6: 1.9 mg/L) but no further significant increase from M6 to M7 (M7: 2.0 mg/L).

The highest GMC and second highest overall increase were observed for serotype 14 (3.2-fold increase), which is present in both PCV13 and PPSV23 vaccines. The maximum antibody concentration at time point M7 was 10.4 mg/L (*P* < 0.0001). However, serotype 14 had the highest baseline level of all serotype-specific antibody concentrations, starting at 4.8 mg/L before the first vaccination. The baseline levels of the other five serotypes ranged from 0.3 to 2.8 mg/L.

The highest increase after sequential vaccination from M0 to M7 was observed for serotype 2 (3.7-fold, *P* < 0.0001), which has an average total antibody concentration of 10.4 mg/L after PPSV23 application. This is the second highest total GMC after serotype 14.

All three serotypes included in the PCV13 vaccine (3, 6A, 14) demonstrated an effect 6 months after the initial vaccination, with 6A exhibiting the smallest increase. However, the mean concentrations remained stable for 7 months without any decline.

Additionally, an increase in antibody concentrations was observed after the first dose for both serotypes included in the first and second vaccinations (3 and 14). However, there was only a minor increase after the second dose (at M7).

In addition to the measurement of serotype-specific immune responses, in this study, a global ELISA test for 23 serotypes was performed on 171 serum samples to assess the overall antibody response against pneumococcal antigens. The test measured global levels of IgG, IgG2, IgM, and IgA, without considering the exact specificity of different serotypes. According to Vidarsson et al., immunoglobulin G-antibody responses to bacterial capsular polysaccharide antigens such as *Haemophilus influenzae* or *S. pneumoniae* are predominantly restricted to IgG2, whereas IgG1 and IgG3 tend to target viral proteins (15).

**Fig 4:**
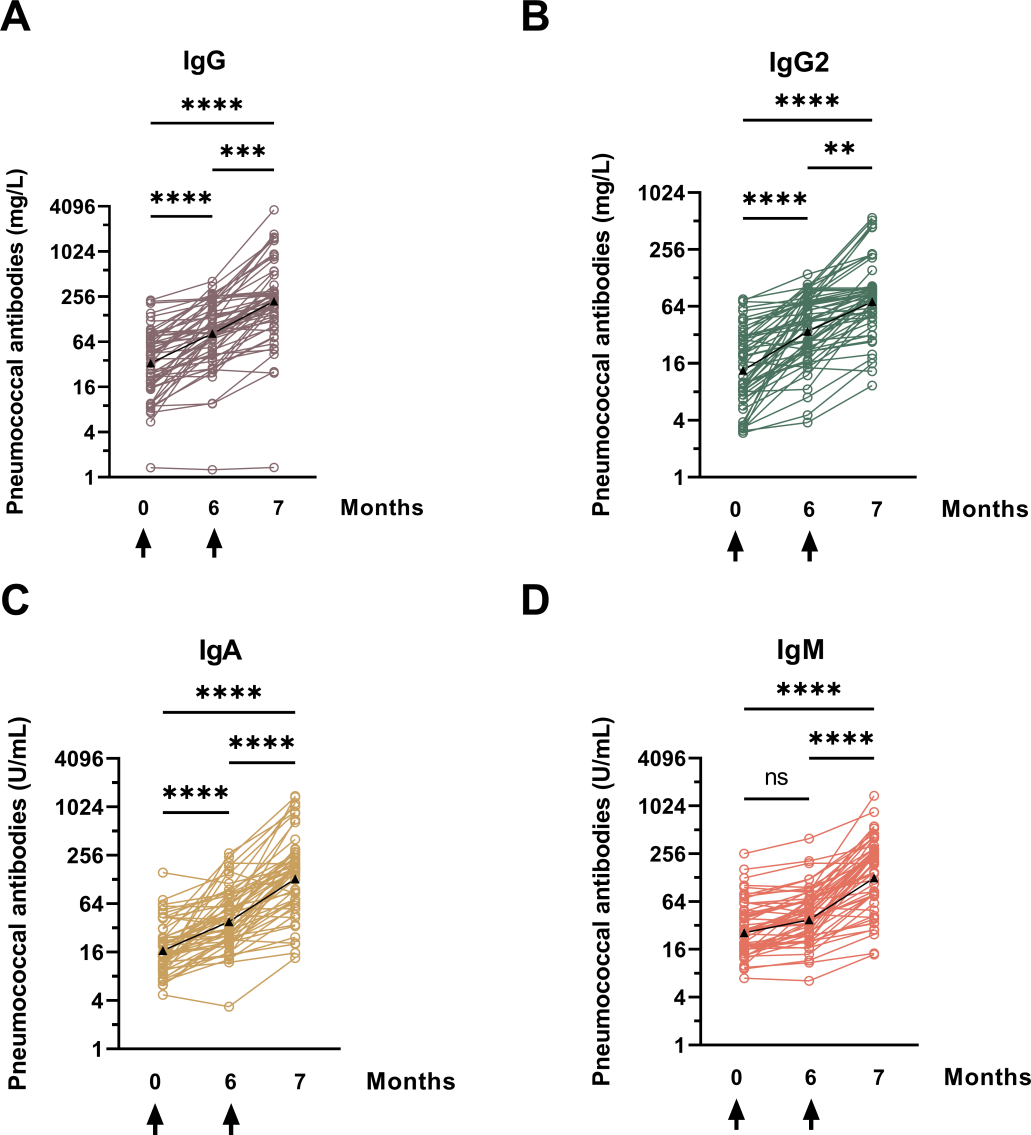
The individual patterns of pneumococcal antibodies in plaque psoriasis patients were analyzed before and after being vaccinated with PCV13 (at month 0) and PPSV23 (at month 6). The vaccination times are marked by an arrow. The results were presented in four panels: panel A shows IgG antibodies, panel B IgG2, panel C IgA, and panel D IgM. The antibody levels were measured by a commercially available ELISA that detects antibodies against 23 different serotypes (global ELISA). The data from 57 psoriasis patients were analyzed using the Kruskal-Wallis test with Dunn’s multiple comparisons test. The results were categorized as not significant (ns), ** for *P* < 0.01, *** for *P* < 0.0005, and **** for *P* < 0.0001.

**Fig 5:**
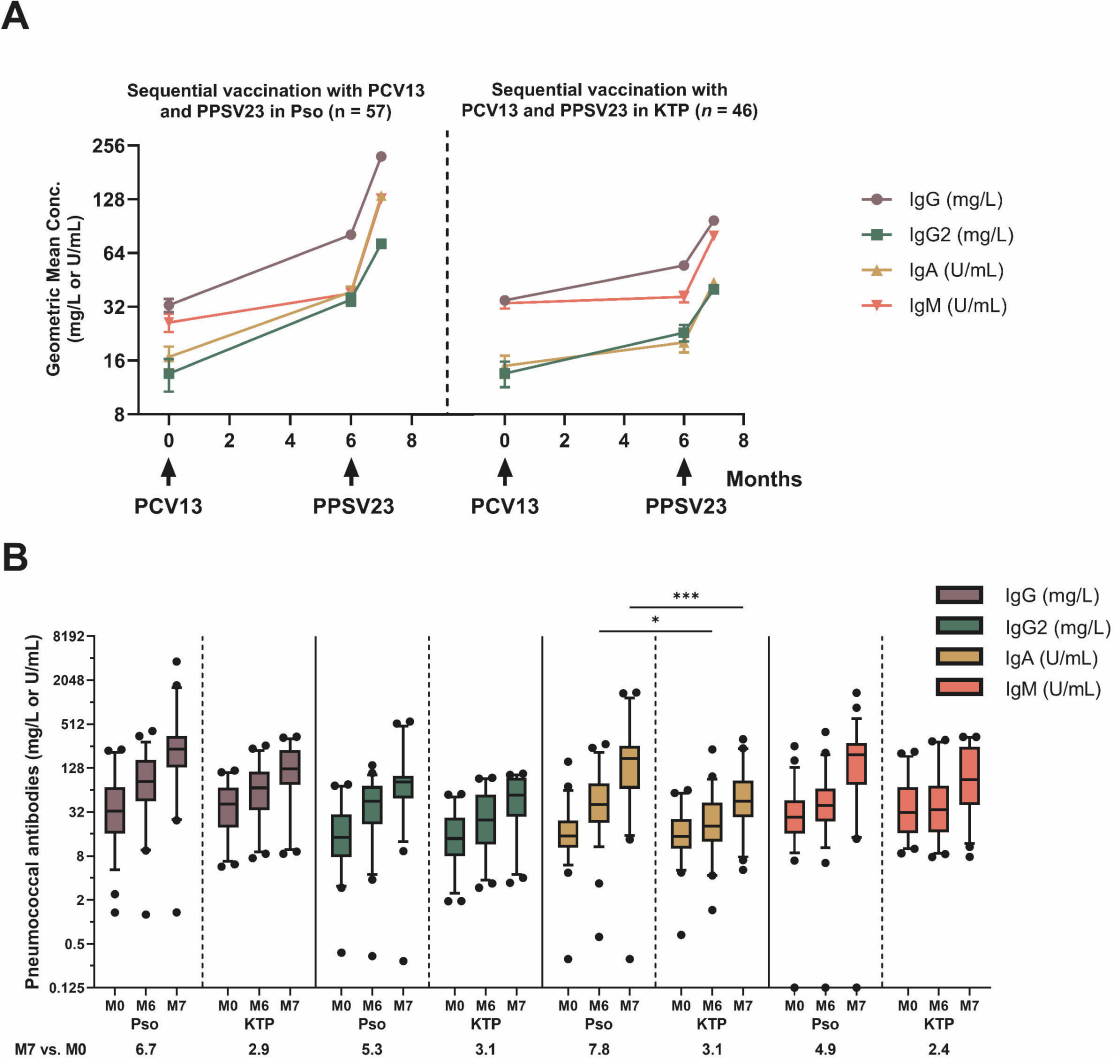
Kinetics of pneumococcal antibodies in plaque psoriasis (Pso) and kidney transplant patients (KTP) as previously carried out in our center (11). The study included patients who were vaccinated sequentially with PCV13 and PPSV23 (month 0 and month 6, respectively). The patients were tested using an ELISA to determine the levels of IgG, IgG2, IgA, and IgM antibodies against 23 different pneumococcal serotypes. (**A**) The results were presented as geometric mean concentration and geometric standard deviation factor. An arrow indicates the time of vaccination. (**B**) The boxplot shows the global antibody concentrations of the different antibody classes of the two cohorts of Pso and KTP. In addition, the ratio of the final to starting GMC for the respective antibody class is given. Comparison between corresponding time points was performed using unpaired Student’s *t* test. * for *P* < 0.05, *** for *P* < 0.0005.

Figures 4 and 5 illustrate that antibody concentrations increase logarithmically over 7 months following sequential vaccination. Different units of antibody concentrations do not allow for a direct comparison of absolute GMC across all four antibody classes. However, over the 7-month measurement period (M0–M7), we observed a significant increase in antibody concentrations for each antibody class, with the highest increase observed for IgA (7.8-fold increase), followed by IgG (6.7-fold increase) (Fig. 5).The increase is rather uniform, with the exception of IgM, which rises slightly after the first vaccination with PCV13 and accelerates after the second vaccination with PPSV23 (M0: 26.2 U/mL, M6: 38.0 U/mL, M7: 128.9 U/mL). Significant increases in IgM antibodies were observed from M0 to M7 (*P* < 0.0001), but the increase was non-significant in the first 6 months. All antibody classes showed a significant increase in GMC between M6 and M7, indicating that vaccination with PPSV23 had a further impact on antibody levels.

The antibody response curves for IgG and IgG2 were almost identical, with the curve for IgG2 at a lower concentration level (IgG: M0: 32.7 mg/L, M6: 81.13 mg/L, M7: 222.3 mg/L; IgG2: M0: 13.5 mg/L, M6: 35.1 mg/L, M7: 72.0 mg/L). IgA showed the highest overall increase (GMC IgA M0: 16.8 U/mL, M6: 38.77 U/mL, M7: 133.9 U/mL). Stratification of the cohort by age ≤48 or >48 years (median) at baseline showed no significant difference (data not shown).

The global change in antibodies in immunocompromised psoriasis patients can be compared to another study conducted at our university hospital in Essen (11). This study analyzed global antibody concentrations in KTP undergoing calcineurin-inhibitor-based therapy with or without mycophenolic acid (MPA) intake during sequential pneumococcal vaccination. Both patient cohorts were comparably large (*n* = 46 and 57, respectively), received the same vaccination schedule, and had pneumococcal antibody assays performed with the same methods and in the same laboratory.

**Fig 6:**
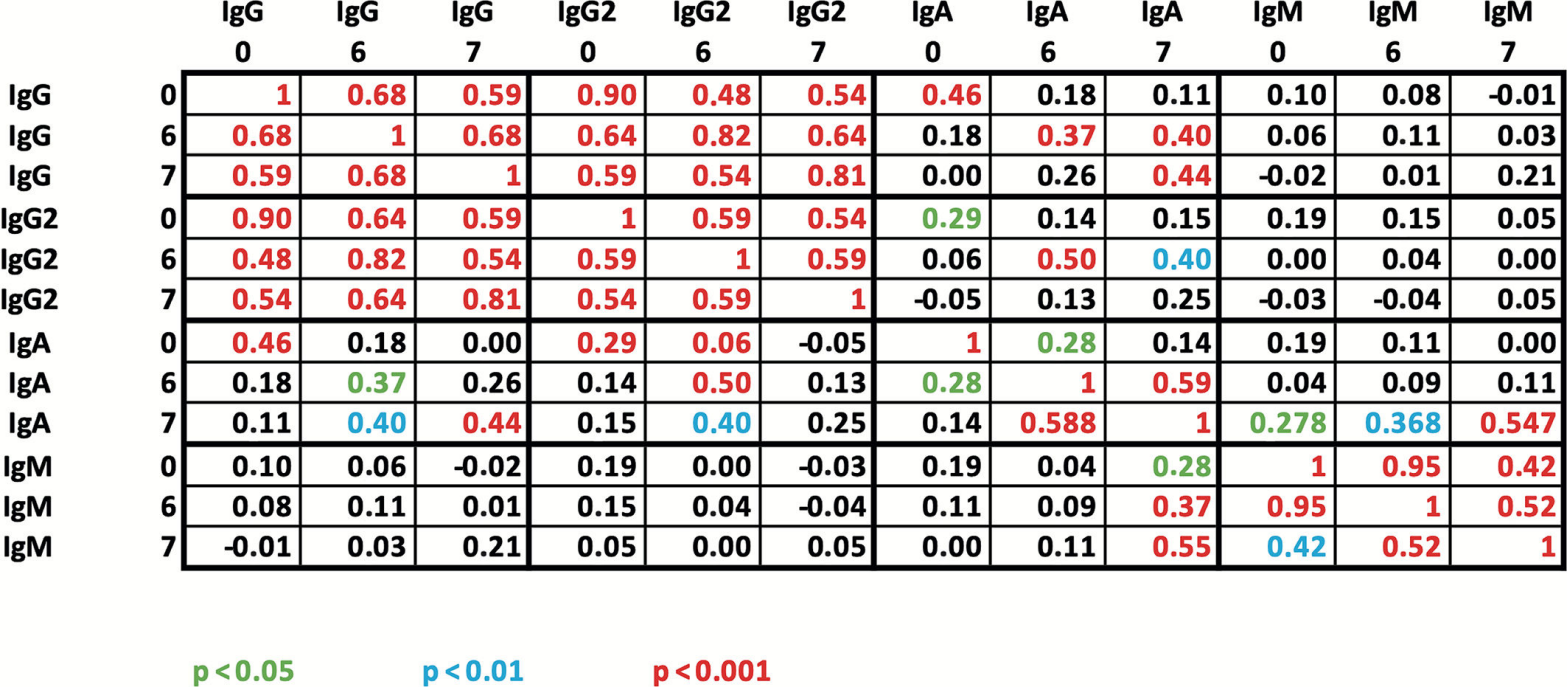
Spearman correlation analysis of pneumococcal antibodies in 57 individuals with plaque psoriasis who had sequential vaccinations with PCV13 (at month 0) and PPSV23 (at month 6). Using a commercially available ELISA that measured antibodies against 23 serotypes (global ELISA), the concentrations of IgG, IgG2, IgA, and IgM antibodies were assessed. The heading’s numbers (0–7) denote the time points (month 0–month 7). Correlation coefficients are represented by the numbers in the table, which are colored according to their level of significance.

Figure 6A illustrates that the initial antibody concentrations are nearly identical for both cohorts. In both test series, the highest initial concentrations were measured for IgG (psoriasis patients: 32.7 mg/L; KTP: 36.49 mg/L). The initial concentration of IgM was slightly higher in KTP compared to psoriasis patients (psoriasis patients: 26.16 U/mL; KTP: 35.12 U/mL). After vaccination, both cohorts exhibited an increase in the concentration of all antibody classes. However, it was found that the increase in GMC after both the first and the second vaccination was significantly lower in KTP than in psoriasis patients despite almost identical initial concentrations. At 6 months, this was especially noticeable for IgM. After vaccination with PCV13, the GMC remained almost unchanged in KTP (M0: 35.12 U/mL, M6: 38.24 U/mL), whereas psoriasis patients showed an increase of almost 12 U/mL in that same period despite the still overall flattest increase compared to other antibody classes. The slopes of antibody concentrations for IgG, IgG2, and IgA as illustrated in Fig. 6A increased homogeneously within the two cohorts. Despite this uniform increase, the overall levels of antibody concentrations were found to be significantly lower in KTP.

The maximum GMC observed at 7 months after sequential vaccination in both studies was for IgG (psoriasis: 222.3 mg/L; KTP: 105.5 mg/L), followed by IgM. Although there was no significant increase after PCV13, the concentration of IgM showed the strongest increase after PPSV23 (M7: psoriasis patients: 128.9 U/mL; KTP: 85.98 U/mL). When comparing the antibody concentrations at baseline with those at 7 months for both groups, KTP consistently showed a lower ratio. The largest difference was observed for IgA, with the GMC increasing 7.8-fold in psoriasis patients compared to only 3.1-fold in the KTP group. This means that the IgA concentrations after the second vaccination in psoriasis patients increased 2.5 times as much as in KTP. The second highest values were measured for IgG (psoriasis patients: 6.7-fold; KTP: 2.9-fold), whereby the GMC in psoriasis patients increased 2.3 times as much as in KTP (Fig. 6B).

The concentration of these four antibody classes was compared to a reference cohort of vaccination-naïve healthy blood donors. The values were derived from two different studies by Schauer et al. and Parker et al. (13, 14). The healthy control cohort consisted of 386 or 73 healthy subjects, respectively (13, 14). Participants were healthy at the time of inclusion and did not suffer from recurrent infections nor did they have any chronic infectious diseases (Table 3). Thus, concentrations in the healthy controls are expected to be even higher after sequential vaccination with PCV13 and PPSV23.

**TABLE 3:** Time course of pneumococcal antibody levels in 57 psoriasis patients vaccinated with PCV13 at month 0 and with PPSV23 at month 6 and comparison of their antibody concentrations with a healthy reference cohort^*a*^

Time point	Antibody class							
	IgG		IgG2		IgA		IgM	
	GMC	PRC	GMC	PRC	GMC	PRC	GMC	PRC
Month 0	32.7	42	13.5	39	16.8	33	26.2	21
Month 6	81.1	75	35.1	79	38.8	81	38.0	35
Month 7	222.3	91	72.0	88	133.9	89	128.9	77

^*a*^ GMC (given for IgG and IgG2 as mg/L and for IgA and IgM as U/mL); PRC, percentage comparable to reference cohort. The reference group comprises healthy blood donors (13, 14). Reference values were defined as concentrations ≥43.8 mg/L for IgG, ≥20.5 mg/L for IgG2, ≥21.0 U/mL for IgA, and ≥54.0 U/mL for IgM, respectively.

We compared the antibody concentrations of our study cohort with the mean concentrations of the healthy controls and calculated the percentage of our subjects who reached or exceeded a threshold for healthy controls as defined in the literature (PRC = percentage comparable to reference cohort) (13, 14). The respective thresholds in literature were defined as ≥43.8 mg/L for IgG, ≥20.5 mg/L for IgG2, ≥21.0 U/mL for IgA, and ≥54.0 U/mL for IgM.

Prior to vaccination (M0), 21%–42% of patients achieved antibody concentrations higher than the threshold that was defined in healthy controls. The highest percentages were found for IgG and IgG2, with antibody concentrations above the threshold measured in 88% and 91% of our subjects at 7 months. While the PRC for IgG, IgG2, and IgA already showed a major increase after the first vaccination at M6 (IgG: 75%, IgG2: 79%, IgA: 81%), it was only minor for IgM: after 6 months, only 35% of the subjects reached the threshold of the healthy control group, but only 1 month later (after vaccination with PPSV23), more than twice as many subjects reached this value (77% at M7).

Spearman rank correlation analysis was conducted on the various antibody classes at three different time points. The results, displayed in the table below (Fig. 6), indicate consistent positive correlations within each antibody class (IgG, IgG2, IgA, and IgM). The correlations consistently reached a significance level of *P* < 0.001. This suggests that the baseline concentrations are predictive of antibody concentrations after the first and second vaccination.

The study found strong positive correlations between identical antibody classes at different time points. Specifically, the IgG and IgG2 antibodies showed the strongest positive correlations, with slightly higher correlation coefficients between baseline and month 6 compared to month 7. The IgM antibodies showed the highest correlation coefficient between baseline and month 6.

Additionally, there was a strong positive correlation between the IgG and IgG2 classes. The correlation of antibody concentrations at months 6 and 7 was similar to that observed within each antibody class. The correlations between IgG and IgG2 were already strong at time point 0, prior to sequential pneumococcal vaccination, and remained almost identical after vaccination at months 6 and 7. This suggests that the relationship between IgG and IgG2 is stable and not significantly influenced by vaccination. No significant positive correlations were found between IgG subclasses and IgM (*P* > 0.05). However, moderate correlations were observed between IgG subgroups and IgA at the same measurement times (correlation coefficient between 0.25 and 0.50).

### Correlation between clinical patient characteristics and pneumococcal antibody concentrations

**Fig 7:**
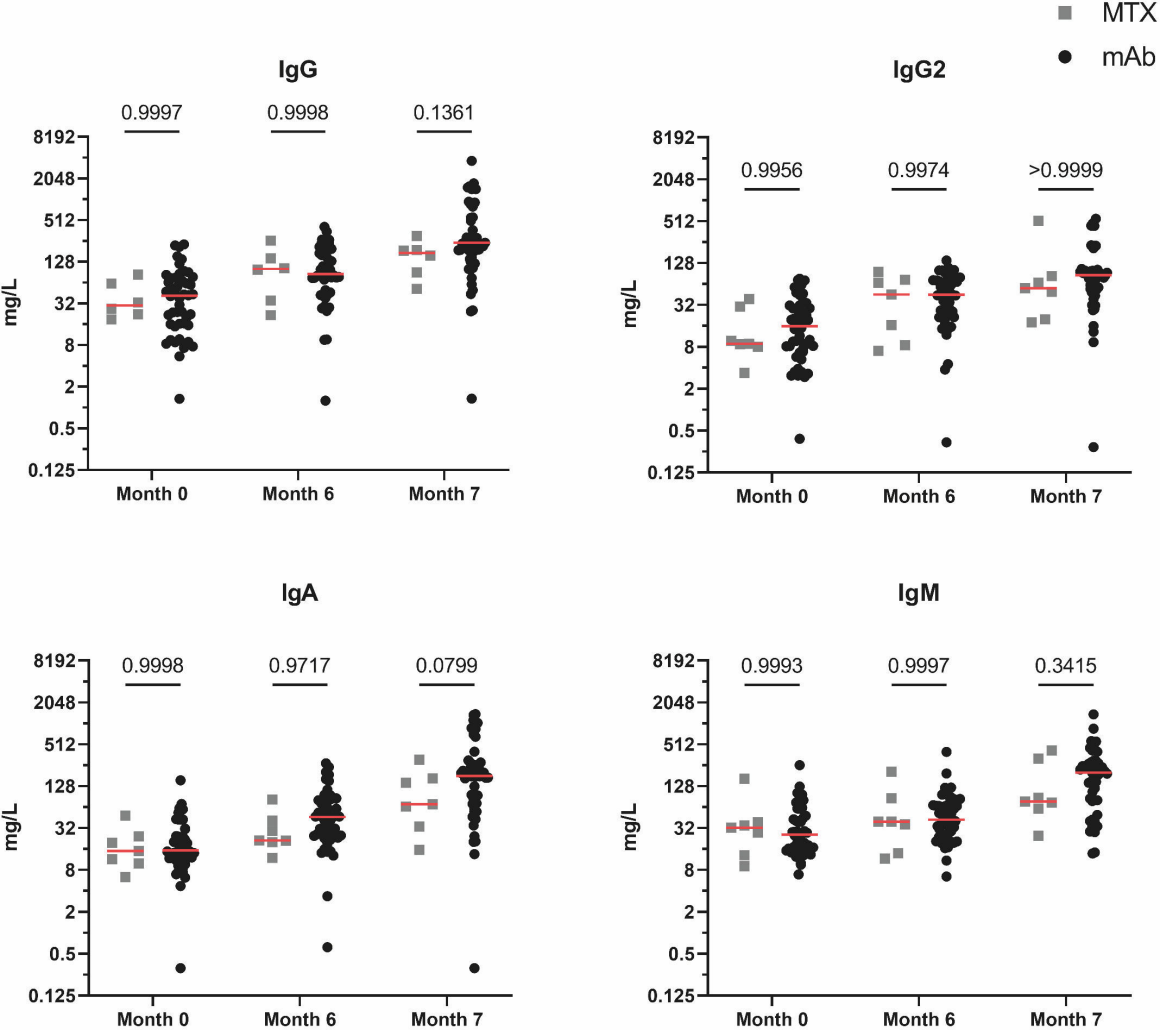
Comparison of global IgG, IgG2, IgA, and IgM antibody responses divided by treatment group (biologicals [mAb] vs methotrexate [MTX]). Mann-Whitney *U* test was performed for comparison between groups, and *P* values are given as exact numbers. Red lines indicate median values.

The study investigated the impact of different systemic anti-psoriatic therapies, including MTX, TNF-α inhibitors (adalimumab/infliximab/etanercept/certolizumab), and interleukin inhibitors (ustekinumab, tildrakizumab, guselkumab, secukinumab, and ixekizumab), on global antibody responses for IgG, IgG2, IgM, and IgA. The results showed a consistent increase in antibody levels across all therapy groups over a period of 7 months. Due to the strong similarity in their mode of action, we combined data on patients treated with TNF-α and interleukin inhibitors (= biologicals) (Fig. 7).

Figure 7 shows that the group treated with biologicals consistently had slightly higher antibody concentrations across all antibody classes at month 7 compared to the MTX group. At baseline and month 6, the antibody concentrations did not show any difference between patients treated with biologicals and MTX.

**Fig 8:**
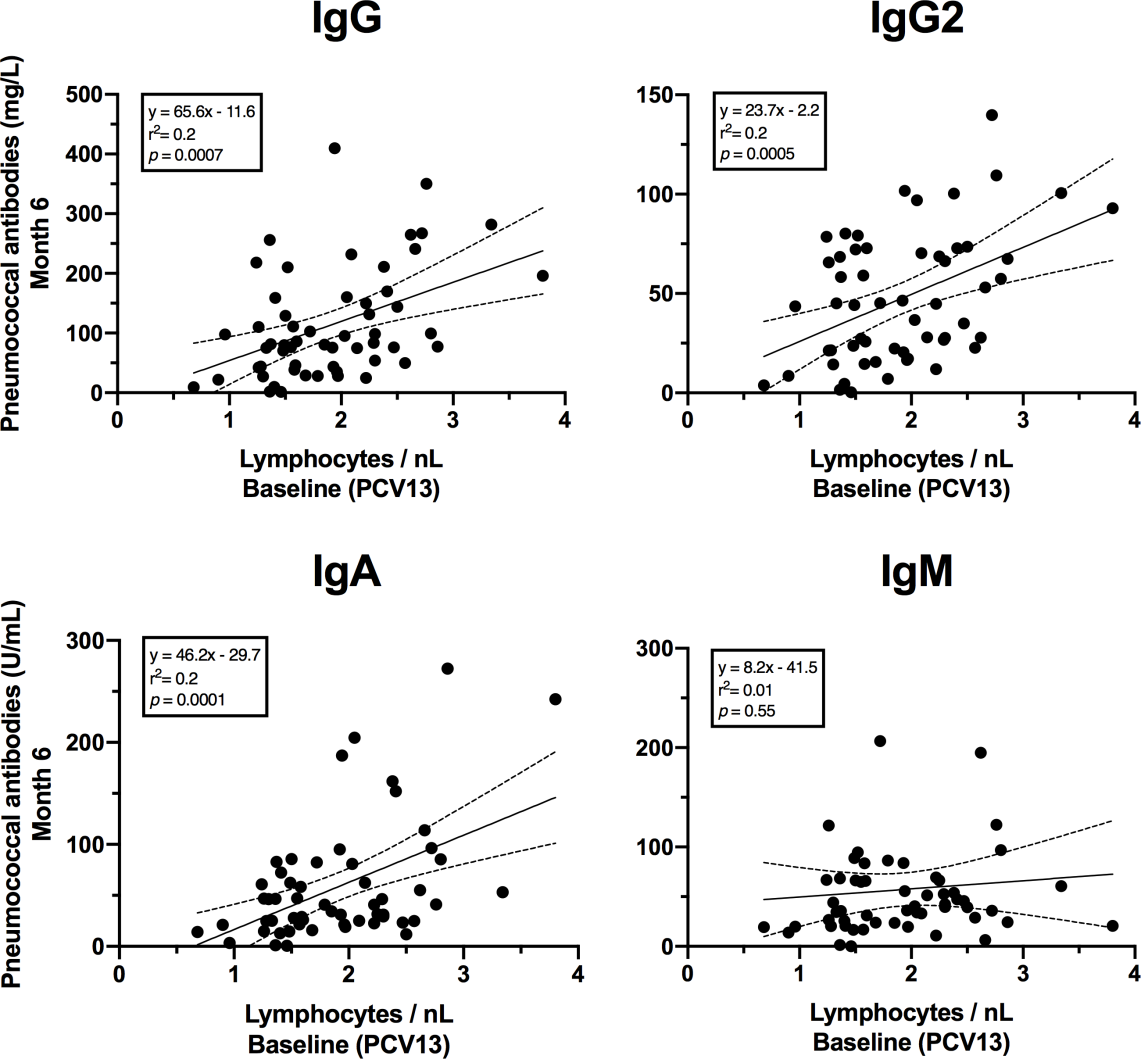
Correlation between baseline lymphocyte numbers and pneumococcal antibodies 6 months after PCV13 vaccination. The 95% confidence intervals of linear regression are indicated by dashed lines. Linear equation, *r*^2^, and *P* values are given for each immunoglobulin type.

In addition, a significant positive correlation was found between lymphocyte numbers at baseline and pneumococcal antibody concentrations at month 6 after vaccination with PCV13 (Fig. 8). This correlation could be detected for the immunoglobulin classes IgG, IgG2, and IgA. However, a correlation between lymphocyte numbers at month 6 and subsequent vaccination response could not be found for PPSV23.

## DISCUSSION

The current study addressed the serological immunogenicity and safety of sequentially immunizing psoriasis patients with PCV13 and PPSV23 under systemic anti-psoriatic treatment. We were able to show that this vaccination schedule elicited a serological response, as evidenced by a considerable increase in both global and serotype-specific antibody levels. No progress of psoriasis or related illnesses was noticed in our cohort over the 26-month follow-up period following the initial immunization (with PCV13). Our data suggest that the sequential administration of both vaccines to our cohort is safe from an immunological point of view.

The patient age in the study ranged from 18 to 79 years, with a median of 48 years. For patients younger than and older than 65 years, the Centers for Disease Control and Prevention (CDC) suggests different time intervals between vaccinations. The interval between PCV13 and PPSV23 vaccination for younger individuals varies according on their specific risk factors. However, the interval should not be less than 8 weeks. For healthy adults aged 65 or older, the CDC suggests a minimum interval of 1 year, but in the case of immunodeficient transplant patients, it can be shortened to 8 weeks (16). In our study, we followed the current vaccination recommendation for risk groups by the German advisory board for vaccination (STIKO), which suggests a vaccination interval of 6 months (17).

Our analysis places more emphasis on the relative change in antibody concentrations due to the well-known issue of the absence of robust thresholds in pneumococcal vaccination trials. There is only modest information on general and serotype-specific anti-pneumococcal antibody concentrations in healthy adults before and after vaccination, and thresholds for protective antibodies are not yet well established. Researchers who have conducted studies to determine a level of protecting antibodies generally agree that long-term protection is indicated by a pneumococcal serotype-specific concentration of more than 1.3 mg/L 1 month following vaccination (18, 19). In older publications, it is assumed that an appropriate response is fourfold or more compared to the baseline value for a given serotype (20). This is also due to the high variance of tests from different laboratories and reference laboratories and the lack of a clear cutoff concentration (19). As previous evaluations of vaccination studies have shown, reactions differ considerably both between individuals and between serotypes within the same person. The fact that the 1.3 mg/L threshold does not do justice to the evaluation of all serotypes is shown, for example, by the comparatively high mean baseline values of the serotype-specific antibodies. The concentration of antibodies against three out of six tested serotypes in vaccine-naïve psoriasis patients already at baseline exceeded this threshold. In most patients with a baseline concentration of more than 1.3 µg/mL, vaccination can achieve a twofold increase in titer, which is also considered as appropriate (21). For all others, an adequate vaccination response is an antibody concentration after immunization of more than 1.3 g/mL or a fourfold increase (18). Therefore, we also compare the antibody concentrations after vaccination with baseline values, as it was done in the phase III clinical trial evaluating the safety and immunogenicity of PPSV23 (12).

When assessing the serotype-specific immune response, it is striking that serotype 3 neither exceeds the geometric mean threshold of 1.3 mg/L nor that a more than fourfold increase in concentration compared to the initial value is observed. With a mean peak value of 0.9 mg/L, plaque psoriasis patients had a higher immune response than kidney transplant recipients (0.6 mg/L) but nevertheless only slightly less than half of what was achieved in a healthy reference cohort after a single vaccination (11, 22). Under these aspects, vaccination success for serotype 3 appears to have occurred in only a few patients. This is particularly problematic, considering that serotype 3 has become one of the most prevalent serotypes in adults in Europe (23). That this is not just a problem in our immunocompromised cohort is shown by the fact that since the introduction of the pediatric PCV13 vaccination programs, declines in serotype 3 have been less than for any other vaccine serotype (24). Our result is thus in line with numerous other studies that confirm that vaccination against serotype 3 has a measurable but still unsatisfactory effect (23, 24). It appears that some serotypes are more antigenic than others, e.g., serotypes 14 and 2. As supposed from the results of previous studies in healthy and immunocompromised vaccinees, serotype 14 achieved the highest absolute concentration of antibodies after vaccination, with a mean of 15.5 mg/L (11, 20, 25, 26). Despite the fact that serotype 2 is only contained in PPSV23, it had the highest increase in the serotype comparison with a 3.7-fold increase after vaccination in plaque psoriasis patients. This is not surprising, as a larger-scale evaluation of the serotype-specific antibody response of PPSV23 has already shown that serotype 2 has the second highest seroconversion rate among all 23 serotypes (26). At a geometric mean fold increase of less than twofold, serotype 6A, which is only contained in PCV13, was the poorest. In contrast to the serotype-specific immune response, global anti-pneumococcal antibodies consistently achieved a more than fourfold increase in GMC at month 7 compared to baseline. In general, with the exception of a few cases of less immunogenic serotypes, sequential vaccination in chronic plaque psoriasis patients can be assumed to be a very effective vaccination strategy.

The current results on psoriasis patients were compared with data from a previous pneumococcal vaccination study in KTP at our center (11). Both the vaccination scheme and the methods are identical in these two studies. In contrast to the KTP cohort, where a strong booster effect of serotype 14 by PPSV23 was observed, this effect was significantly lower in psoriasis patients due to the already strong vaccination response to PCV13. In the case of serotype 3, which induced a poor response anyway, the vaccination response had a comparable booster effect to that of KTP. Plaque psoriasis patients achieved higher antibody concentrations in absolute numbers than KTP for all tested serotypes. This can be explained by the fact that the patient cohorts differ in immunosuppressive medication and the extent of immunosuppression. In principle, drug-induced lymphopenia can affect the immune system regardless of the cause. Since the immune response to vaccination, including pneumococcal vaccination, is largely controlled by lymphocytes, a reduced number or function of lymphocytes could theoretically affect the effectiveness of the immune response (27). When looking at the global immunoglobulin levels compared to kidney transplant patients at month 7, it is noticeable that plaque psoriasis patients have 1.4 to 2.8 times higher antibody concentrations (11). The large difference in the IgA antibody response is particularly noteworthy. KTP had a maximum GMC of 46.5 U/mL 1 month after the second vaccination and psoriasis patients of 133.9 U/mL (*P* < 0.0005) (11). In addition, 6 months after the first vaccination with PCV13, there was already a significant difference in the total IgA concentrations (*P* < 0.05). Even if the relevance of an IgA vaccination response after pneumococcal immunization still requires further research, it can be assumed that IgA as the predominant immunoglobulin isotype in the upper and lower respiratory tract with other effector mechanisms than IgG has a considerable protective effect (28). In saliva, however, mainly polymeric, stabilized secretory IgA is found (29). Whether the IgA values measured in the serum also reflect the actual secretory IgA concentrations in the mucosal areas would be an interesting approach for further studies. A differentiation between the vaccines PCV13 and PPSV23 and their influence on secretory IgA would be interesting here, as PPSV23 can only influence T-cell-independent IgA production (30). However, this only accounts for around a quarter of total secretory IgA production (29). The difference between psoriasis patients and KTP can also be seen in comparison to healthy blood donors. For example, while only 83% of KTP achieved IgG antibody concentrations exceeding a threshold established in the healthy controls, in this study, the percentage was 91%. This difference is most likely explained by the broader immunosuppression and therapy regimens that include MPA. MPA was previously shown to worsen responses to other vaccinations, such as anti-SARS-CoV-2 mRNA vaccines (31). For moderate-to-severe plaque psoriasis, the German guideline generally recommends conventional drugs such as MTX as first-line therapy. Usually, biologicals are used as second-line treatment and, in special cases (e.g., PASI > 20, significant impairment of quality of life, or rapid disease progression), also as first-line therapy (32). In previous vaccination trials, MTX was shown to have a significant impact on vaccine responses in patients with plaque psoriasis (6, 33, 34). Due to the high incidence of side effects associated with MTX treatment, immunosuppressive therapy regimens are increasingly shifting from MTX to targeted interleukin inhibitor therapies (35). For this reason, the group of patients treated with MTX that we recruited was significantly smaller than the group of patients under therapy with biologicals. In accordance with the abovementioned studies (33, 34), patients treated with MTX tended to mount lower antibody responses compared to patients under treatment with biologicals. Unfortunately, our research does not include a sufficient number of individuals who were treated with MTX, which would significantly improve the strength of these data. In previous studies, the effects of TNF-α inhibitors have been inconclusive and associated with both better and worse responses to PPSV23 vaccination than treatment with other immunosuppressants (36–38). In a larger meta-analysis analyzing the influence of immunosuppressive therapy, MTX showed a more negative effect than TNF-α inhibitors on the seroconversion rates after PCV13 vaccination (38). However, the effect was exactly the opposite for the PPSV23 vaccine, where TNF-α therapy led to a poorer immune response (38). Other biological therapies such as the IL-17A antagonist ixekizumab also proved to be non-inferior in terms of immune response to pneumococcal vaccinations (39). In individuals with moderate to severe psoriasis, the monoclonal IL-12 and IL-23 antibody ustekinumab also does not seem to negatively impact the immune response to T-cell-dependent/independent vaccinations (40).

In summary, there are contradictory statements about the effect of the various immunosuppressants in individual studies. Interleukin inhibitor therapy appears to have little or no overall effect on vaccine response, as was the case in our results in comparison with healthy controls. Overall, there was no discernible difference between the immunosuppressive strategies due to the small subgroups. Either way, it can be concluded that sequential vaccination is beneficial irrespective of personal medication.

From studies with other immunocompromised patients, such as HIV-infected patients, KTP, and hematopoietic stem cell transplant patients, we know that stable antibody concentrations can be expected even after five or more years if there is a good initial vaccination response (41–43). Given the comparatively less intense drug-induced immunosuppression, a correspondingly good long-term response can be assumed for patients with psoriasis. This long-term protection is based both on protective serum antibody levels and immunological memory in the form of antigen-specific memory B cells (44). However, follow-up studies are needed to understand how many specific anti-pneumococcal memory B cells were induced by sequential vaccination in immunocompromised patients and whether or how long serologic memory is maintained.

A complex coordination between cells across the body is necessary for the immune system to react to a vaccine. The amount and quality of cells present at time of vaccination have a significant impact on the immunological response to the vaccine (45). In our analysis, we demonstrated a positive correlation between antibody concentrations 6 months post-vaccination with PCV13 and the baseline lymphocyte concentration for IgG, IgG2, and IgA. However, such correlation was not observed with the PPSV23 vaccine. In addition to investigating the memory response, future vaccination studies could provide valuable insights by examining the phenotype of B and T cell subsets in the peripheral blood of patients at the time of vaccination.

In conclusion, patients with moderate to severe plaque psoriasis under systemic therapy can achieve protective antibody concentrations under systemic therapy after sequential vaccination according to the literature. Vaccination success varies depending on the pneumococcal serotype. The type of immunosuppressive therapy had no significant effect on the response to vaccination. However, we saw a trend toward lower antibody concentrations in patients with MTX treatment. Overall, a significant increase in antibody concentrations can be observed as a result of the sequential vaccination so that a satisfactory vaccination response can be assumed.

## Data Availability

The data presented in this study are available on request from the corresponding author. The data are not publicly available due to privacy restrictions.
